# Supplementary material for: Are carbon emissions trading and green financial instruments synergistic? -Comprehensive quantitative research based on content analysis
Source: PLoS One. 2024 Mar 7;19(3):e0298601. doi: 10.1371/journal.pone.0298601 (PMC10919601; doi:10.1371/journal.pone.0298601)
Supplement: S1 Data — (ZIP) [file pone.0298601.s001.zip › Data/地级市Z_F工作报告.zip!/╣≤╓▌/╫±╥σ╩╨/└┤╫╘╣1⁄2╓┌║┼í╛┬φ┐╦╩2╛▌═°í┐_2007.docx]

各位代表：

现在，我代表市人民政府向大会作工作报告，请予审议，并请各位政协委员提出意见。

一、过去四年工作回顾

过去四年，市人民政府在省委、省政府和市委的领导下，在市人大和市政协的监督支持下，坚持以邓小平理论和“三个代表”重要思想为指导，认真贯彻落实科学发展观，紧紧围绕“三化一强”战略，抢抓机遇，加快发展。通过全市广大干部群众的共同努力，“十五”计划全面完成，市二届人民政府确定的预期目标圆满实现，“十一五”规划开局良好，经济社会持续快速协调健康发展。

国民经济持续增长，综合实力显著增强。2006年，全市生产总值预计完成468.1亿元,比上年增长12.6%，四年年均增长12.7%，快于全省1.5个百分点，连跨300亿元、400亿元两个台阶；人均生产总值6244元，年均增长11.8%；财政总收入72.23亿元，年均增长24%，其中地方财政收入26.88亿元，年均增长18%。

“三化”建设稳步推进，产业结构不断优化。农业产业化经营“五突破”成效明显。畜牧业、竹业、药业等主导产业快速发展，“七大产业航母”基本形成，“六个一百”工程进展良好。农业增加值年均增长6.26 %，粮食总产量稳定在300万吨左右，肉类总产量年均增长10.83%，畜牧业产值占一产的比重达到40.6%，农业基础地位进一步夯实，首创的以富、学、乐、美“四在农家”为载体的社会主义新农村建设，在全国产生了广泛影响进而推广。工业化进程加速。“六大基地”已具雏形，“六个百亿行业（企业）”发展顺利，“六大标志工程”稳步推进，重点工业项目技改加快,四年累计完成技改投资118.47亿元。工业经济总量不断壮大，工业增加值2006年预计达到170亿元，年均增长21.04%，工业对财政的贡献率达到 80 %以上，工业经济主导地位日益巩固。城镇化步伐加快。城镇规划、建设和管理同步加强，累计完成城镇建设投资150多亿元，城镇化率达到33.4%，新建了汇川区，中心城区承载100万人口的框架基本确立。实施了遵义会议陈列馆、红军烈士陵园、杨柳街历史街区保护等纪念体系建设工程；图书馆、博物馆等文化建设工程；汇川大道、官井隧道、洗马路和九节滩、高桥、遵湄路口三大入口改造等交通疏解工程；湘江河污水截流、10万亩风景林、中华路街心花园等环境整治工程；中心城区小街小巷整治、饮用水源保护、垃圾无害化处理厂、医疗特殊垃圾处置厂、仁怀赤水习水污水处理厂及垃圾填埋场等环境保护工程。城镇功能日趋完善，人居环境明显改善，山水园林城市特色凸显，先后荣获“全国绿化模范城市”、“国家园林城市”、“中国优秀旅游城市”、“全国无偿献血先进城市”等殊荣，创建“国家卫生城市”通过了国家爱卫办技术考核。第三产业发展提速，房地产、旅游、通信等新兴服务业发展迅猛。2006年，全社会消费品零售总额达到140亿元，年均增长 14.1%；旅游综合收入达 35.5亿元。三次产业结构由2002年末的31.19：36.57：32.24调整为21.5∶43.1∶35.4，产业结构进一步优化。

投资拉动效果明显，西部开发成效显著。抢抓国家西部大开发机遇，“五年打基础”的目标提前实现。四年累计完成全社会固定资产投资709.5亿元，年均增长20.6%，投资拉动经济增长年均7.5个百分点以上，城乡面貌发生了巨大变化。交通基础设施建设取得突破性进展,崇遵高速公路建成通车,贵遵高速公路实施改造，白腊坎至茅台高速公路及习水至桐梓新站、湄潭至余庆、绥阳至道真二级公路开工建设，完成了中心城区6条出口二级公路和14条县际公路改造，“四纵三横一联线”骨架公路网基本建成；建成通乡油路97条2112公里、通村公路642条6008公里，基本实现乡乡通油路、88%的行政村通公路，农村公路建设“三年目标”基本完成，公路总里程超过2万公里；完成了乌江、赤水河航道整治和码头建设，建成64个乡镇客运站，全市公路水路网密度、等级、通达深度、通行能力显著提高。农村基础设施建设加强，水利渠系配套、中低产田土改造、农业机械化综合运用、防灾救灾、农村人畜饮水和城镇供水等工作扎实有效，遵义灌区、湄凤余灌区工程进展顺利。能源建设取得新突破，习水火电厂、鸭溪电厂建成投产，乌江电站扩容如期完成，构皮滩电站建设推进顺利，“西电东送”能源基地初具规模。生态建设成效显著，四年累计完成退耕还林292万亩，森林覆盖率达44%以上。义教工程、危改工程、乡镇卫生院等农村社会事业发展项目全面推进。

改革开放深入推进，发展活力明显增强。经济结构战略性调整取得明显成效。大中型企业改制、分离企业办社会取得重大进展，优势企业成功上市，市、县属国有工业企业改革基本实现“两个100%”，国有资产监督管理体制逐步理顺，经营状况得到改善，组建了市国有资产投融资经营管理有限责任公司。非公有制经济发展快速，年均增长20%以上。全面取消了农业税，农村税费制度、粮食流通体制改革取得阶段性成果，农业“一免三补贴”及农村义务教育阶段“两免一补”等惠农政策全面落实。行政审批制度改革取得新进步，率先在全省建立了“一站式”服务机构——政务服务中心，社会各界反映良好。投融资体制、社会保障制度、公共财政体制、农村金融体制、事业单位人事制度、公用事业等各项改革有序推进。投资环境明显改善，对外开放水平进一步提高。多形式搭建招商引资平台，全方位开展区域经济文化交流与合作，成功举办了“中国民营企业家遵义行”、纪念遵义会议暨红军长征胜利70周年、中国政府文华奖第四届、第五届全国青少年杂技大赛等系列重大活动。招商引资成效显著，四年累计到位资金214.9 亿元，年均增长44.2%；四年累计完成进出口总额2.75亿美元，年均增长22.3%。

社会事业全面进步，人民生活明显改善。各类教育协调发展，“两基”攻坚目标提前一年实现，“普九”人口覆盖率达100%，教学质量明显提高；高中教育发展迅速，建成省级示范高中9所，入学率从2002年的28%上升到2006年的33.5%；师范资源整合步伐加快，成功组建了遵义师范学院、遵义职业技术学院和遵义医药高等专科学校，教育布局、结构更趋合理。科技创新能力不断增强，科技成果向现实生产力转化加快，科技贡献率达到40%。人口与计生事业不断创新发展，继续保持全省领先位次，在全国首创了计划生育利益导向机制，优质服务水平逐年提高，符合政策生育率从2002年的88%提高到2006年的97.2%，人口自增率由2002年的9.5‰下降到 2006年的5.55‰，低生育水平继续巩固。医疗卫生服务网络进一步建立健全，公共卫生服务体系初步建立，医疗救治能力、预防控制能力、卫生执法监督能力、突发公共卫生事件应急处理能力增强，成功防范了“非典”和“禽流感”。新型农村合作医疗试点惠及4县（市）200多万农民，人民群众健康水平不断提高。文化、广电事业进一步繁荣，文化基础设施建设加强，累计投入10亿元建设文体设施，“依依山水情”获得了国家舞台艺术精品工程，文化信息共享工程作为试点在全国推广，广播、电视综合覆盖率分别达到81.3%和89.5%，在全省率先启动数字电视整体平移工程，开通了“中国遵义”门户网站群与公务员办公业务资源网。城乡体育设施日臻完善，群众性体育活动蓬勃开展，竞技体育取得新突破。

人民生活水平进一步提高。城镇居民可支配收入、农民人均纯收入分别达到8943元和2445元，年均增长8.6%和5.4%，分别高于预期目标3.6和1.4个百分点。新农村建设稳步推进，完成“四在农家”创建点2500多个，惠及28万户100多万农民群众。扶贫开发成效显著，四年脱贫12.45万人。“四民社区”创建成效明显，新型文明和谐社区工作扎实推进。就业工作取得新进步，四年新增城镇就业岗位15.98万个，城镇登记失业率控制在4.5%以内。社会保障体系不断健全，7万城镇居民和4万农民享受最低生活保障，12.3万名农村特困群众纳入定期救助范围。“十件实事”顺利完成。“平安遵义”创建活动深入开展，社会治安综合治理不断加强，社会持续稳定；“四五”普法和依法治市工作成绩显著，荣获“全国法制宣传教育先进城市”称号，三次蝉联全国社会治安“平安杯”优秀地市奖。突发公共事件应急管理和处置能力不断提高，安全生产连续4年实现事故起数和死亡人数“双下降”。行政监察、审计监督不断加强，政务公开成效明显，效能建设深入推进，廉政建设扎实有效。积极支持驻遵部队建设，重视发挥驻遵部队和民兵预备役人员参与地方经济建设的作用，双拥工作成效显著，四次荣获“全国双拥模范城”称号。救灾救济、优抚安置、民族宗教、水库移民、外事侨务、国防教育、人防战备和档案、残疾人、妇女儿童、老年人等事业均取得长足进步。全市政治安定、社会稳定、人民安居乐业。

各位代表，过去的四年，是我市经济社会发展最快、效益最好、人民群众得实惠最多的四年。四年来的实践为我们积累了宝贵的经验：一是必须求实务快科学发展。始终坚持以经济建设为中心，把发展作为第一要务，坚持以重点带动全局，积极主动贯彻国家宏观调控政策，努力扩大投资，积极促进消费，实现又快又好发展。二是必须创新思路开拓前进。始终立足遵义实际，强力实施“三化一强”战略，创新发展思路和工作方式，完善配套政策和措施，推动经济结构不断优化升级，着力转变经济增长方式，提高发展质量。三是必须统筹兼顾惠及各方。始终坚持以人为本，促进经济、政治、文化、社会全面协调可持续发展；坚持以“四在农家”、“四民社区”、“集团帮扶”等为载体，推进城乡共同繁荣，缩小贫富差距，努力实现好维护好发展好人民群众的根本利益。四是必须依靠群众形成合力。始终坚持为人民服务的宗旨，引导好、发挥好人民群众的积极性和创造性，充分尊重基层和群众的首创精神，努力汇聚全社会推动发展的强大动力。五是必须确保稳定优化环境。始终坚持正确处理改革、发展和稳定的关系，认真研究解决人民群众最关心、最迫切的问题，妥善处理人民内部矛盾，扎实推进“平安遵义”建设、机关效能建设和政务公开，努力营造和谐稳定的发展环境。六是必须提高能力强化保障。始终坚持以增强领导发展的能力为重点，以提高各级政府民主、科学和依法行政能力为关键，加强政府行政能力建设，推进政府管理创新，以务实、清廉、高效的政风，激励全市各族人民团结奋斗。

过去四年的艰苦奋斗，为实现遵义经济社会历史性跨越奠定了坚实基础。成绩的取得，是全市人民和社会各界共同努力的结果。在此，我代表市人民政府，向全市各族人民、知识分子、广大干部职工，向各民主党派、工商联、人民团体、无党派人士，向驻遵解放军指战员、民兵预备役人员、武警和消防官兵、公安民警，向历届老领导老同志及关心支持遵义经济社会发展的各界人士，表示衷心的感谢并致以崇高的敬意！

回顾四年的工作，在充分肯定成绩的同时，我们也必须正视经济社会发展中存在的困难和问题：一是经济总量小、人均水平低，结构性矛盾仍较突出，经济增长方式粗放，与实现经济又快又好发展的要求还有差距；二是农业抗御自然灾害的能力较弱，农民增收困难的状况没有根本改变，富余劳动力转移困难与自我发展能力较弱并存，与社会主义新农村建设的要求还有差距；三是区域之间、城乡之间、经济与社会之间发展不平衡的状况没有根本改变，均衡发展矛盾与社会事业相对滞后并存，与全面协调发展的要求还有差距；四是经济与人口、资源、环境不相协调的状况没有根本改变，资源环境压力过大与人均水平偏低并存，与可持续发展的要求还有差距；五是政府职能转变和行政管理体制改革相对滞后的状况没有根本改变，越位、缺位与错位并存，与加强自身建设、推进政府管理创新的要求还有差距。这些问题，我们将在今后工作中深入研究，努力加以解决。

二、今后五年政府的主要工作

指导思想：高举邓小平理论和“三个代表”重要思想伟大旗帜，坚持科学发展观和构建和谐社会重大战略思想，认真贯彻落实即将召开的党的十七大和省十次党代会精神，坚持发展第一要务，坚持“五个统筹”，着力创新发展模式、转变增长方式、调整经济结构、提高发展质量。按照市第三次党代会和“十一五”规划纲要的总体要求，全力实施“三新一强”战略，抢抓发展机遇，扎实推进社会主义新农村建设，加快推进新型工业化发展，着力培育创新型城市，打造文化旅游强市，全面加强经济、政治、文化和社会建设，推进全面小康，构建和谐遵义，建设与遵义知名度和影响力相称的城市，实现经济社会发展新的历史性跨越。

主要预期目标：到2011年，力争地区生产总值达到900亿元以上，年均增长12.5%，人均生产总值达到1300美元以上；规模工业增加值年均增长16%，固定资产投资年均增长15%，社会消费品零售总额年均增长16%，财政总收入和地方财政收入年均增长13%，城镇居民可支配收入和农民人均纯收入年均分别增长8%和6%，人口自然增长率控制在6‰以内，城镇登记失业率控制在4.5%以内，单位生产总值综合能耗下降20%左右,主要污染物排放减少13%左右。

今后五年政府工作的主要任务：

（一）以“四在农家”为载体，以增加农民收入为核心，扎实推进社会主义新农村建设

坚持把“三农”作为重中之重，坚持多予少取放活、工业反哺农业、城市支持农村的方针，以“四在农家”创建为载体，统筹城乡发展，缩小城乡差距，实现农村总体小康。

加快发展现代农业，着力增加农民收入。坚持高产、优质、高效、生态、安全的现代农业发展方向，按照科学的产业布局，突出区域特色，巩固和提高粮食、烤烟、油菜等传统支柱产业，扎实推进“六个一百”工程建设，加强产业化基地建设，进一步提高农业综合生产能力。推行“公司＋专业合作社＋农户”产业化发展模式，大力发展壮大龙头企业，加速发展乡镇企业，加快发展“订单农业”和“品牌农业”，加快农村专业合作经济组织和专业技术协会发展，壮大经纪人队伍，鼓励土地使用权、技术、资金等要素参与产业化经营，培育壮大药、竹、畜、茶等农产品加工企业，推进农副产品加工园区和集聚带建设。加快农业标准化建设，建立健全农业综合配套体系；加大实施“科技增收工程”和“四种工程”力度，普遍推行良种良法，抓好节本增效；加强农作物、畜禽、林木优良品种繁育、引进、试验、示范和推广，推进种子产业化，逐年提高“四种”自给率；着力提高畜牧养殖效益，加快建设生态畜牧强市。加快农村现代流通体系建设，大力实施“新网工程”，改善农村购销环境，活跃农村市场。以培养新型农民为目标，加强培训，外输内转，加快发展劳务经济。努力建立农民增收的长效机制，促进农民家庭财产普遍增加。

深化“四在农家”创建活动，着力增强农村发展能力。坚持以“四有五通三改三建”为着力点，以“富学乐美”为目标，进一步转变工作思路，逐步实现创建重心的“三个转移”，继续推进“四在农家”创建，扩大惠及面，力争80%以上农户受益。加强镇村建设规划，积极整治镇村环境，提高农村文明程度。加强生态环境建设和保护,继续实施以退耕还林(竹、茶、药等)、天然林保护为重点的林业生态重点工程、沼气工程、小流域综合治理水土保持工程、小水电代燃料工程，启动石漠化综合治理工程，巩固和扩大退耕还林成果,到2011年，全市森林覆盖率达48%以上。大力发展农村教育、卫生、文化、广播电视、体育等社会事业，促进农村经济社会协调发展。深入开展“五下乡”活动，充分发挥现代远程教育网络作用，丰富农民群众文化体育生活，全面提高农民思想道德、科学文化和健康素质，培养和造就新型农民。

推进农村综合配套改革，着力构建农村社会保障体系。按照“三改革一化解”的要求，巩固农村税费改革成果，推进县乡财政管理体制、乡镇机构、义务教育经费保障机制、林业和水利产权制度等综合改革，进一步减轻农民负担、激发农村发展活力。稳定家庭承包责任制，搞活土地使用权流转，大力发展科技、流通、农机等农业社会化服务体系，不断提高农业生产装备水平。严格土地管理，健全土地征用办法和补偿机制，实现耕地总量动态平衡。全面推行以新型农村合作医疗为主要形式的农村医疗保障制度，完善以“五保户”、“低保户”、困难群体为主要对象的农村最低生活保障制度，积极探索建立失地农民养老保险制度和农民工医疗保险、工伤保险制度，使改革发展新成果惠及更多农民。加大新阶段扶贫开发力度，继续实施集团帮扶、整村推进、易地扶贫搬迁和农村茅草房危房改造，重点帮助贫困群众改善基本生产生活条件，拓宽基本增收门路，提高基本素质，确保农村贫困人口稳定脱贫，力争到2011年使80%的农村人口过上小康生活。

（二）以推动发展和创新为主线，以转变增长方式为核心，加快推进新型工业化

以大项目为支撑，以发展和创新为主线，以循环经济为载体，坚定不移地走科技含量高、经济效益好、资源消耗低、环境污染少、人力资源得到充分发挥的新型工业化道路。

优先发展特色优势产业，培育壮大新型支柱产业。按照科学发展观和国家产业政策的要求，集中力量加快发展有遵义特色、有比较优势的产业。着力建设工业“六大基地”，建成以构皮滩电站为代表的“西电东送”能源基地、以铝钛为代表的有色金属基地、以20万吨竹浆林纸一体化为代表的化工基地、以茅台酒为代表的食品加工基地、以江南航天集团为代表的机电制造业基地和以忠庄药业园区为代表的药业基地。着力培育能源、铝业、化工、新材料、机电、食品年销售收入“六个百亿行业（企业）”，积极支持茅台酒集团、遵义碱厂率先成为销售收入上百亿元的大企业（集团），形成产业配套完善、技术领先、质量一流的产业集群。着力建成赤水20万吨竹浆林纸一体化、遵义80万吨氧化铝、习水二郎火电厂、桐梓火电厂、桐梓煤化工、绥阳氯碱循环经济型重化工“六大标志工程”。

加快推进企业技术创新，改造提升传统支柱产业。坚持以信息化带动工业化，加强信息化建设和信息资源的整合利用，鼓励企业自主创新，努力提高生产技术和工艺水平。加大技改投入力度，逐步建立以政府投入为引导、企业投入为主体、银行贷款为支撑、社会投入为补充的企业研发投入机制。加强企业技术中心建设，增强引进、消化、吸收再创新能力，不断提升企业核心竞争力。充分利用高新技术、先进适用技术改造提升传统产业，着力实施一批效益好、技术先进的重点技术改造项目及信息化示范工程，重点提升我市在全国有比较优势的机电制造业、原材料加工业、化工和食品加工业的技术水平。支持061基地加快民品开发，巩固提升以机械制造、电子信息、汽车配件、冰箱等为主的机电工业，做大做强制造业。大力实施名牌发展战略，以名牌带动优势产业发展，积极推动茅台酒、海绵钛、钢丝绳、聚氯乙烯等现有名优产品做大生产规模，扩大市场份额，着力培育一批具有自主知识产权、附加值高和市场竞争力较强的国家级、省级品牌产品，促进名、优、特、精、新产品上规模、上档次。

大力推行资源综合利用，推动工业增长方式转变。按照“减量化、再利用、资源化”要求，积极推进资源节约、综合利用和清洁生产，大力发展循环经济。按照产业集群发展、企业集中布局、土地集约利用、资源节约使用、污染集中治理的要求，重点发展煤电铝一体化、煤电化一体化、煤及煤化工、铝及铝加工、钛及钛加工、竹及竹加工等六大循环经济产业体系。抓好航天高科技、遵义药业、海尔冰箱配套，高桥、龙坑、坪桥、忠庄、董公寺、风华、南白工业示范区，虾子省级农产品加工示范基地，湄潭绿色食品、赤水河谷名酒工业示范区建设，加快国家级新材料、桐梓煤电一体化、绥阳氯碱循环经济型重化工等循环基地建设，使之成为体制机制创新的先导、科技研发的平台，形成资源循环利用的新型发展模式。

（三）以城镇化为载体，以改革开放为动力，努力培育创新型城市

坚持以创新为动力，以机制为保障，提升遵义综合实力，建设创新型城市。

加快推进城镇化和管理创新，努力集聚创新要素。坚持中心城区“南北拓展、东西贯通、完善功能、突出特色”的发展思路，实施大中城市带动战略。以两城区为核心，继续推进旧城改造，开发建设南部、北部两大新区，建设新蒲科教新区，努力打造创意产业园区，着力拓展城市发展空间。以建设承载200万以上人口的中心城区为远期目标，加快城市总体规划修编，高起点、高标准规划和建设城市，培养增长极，提升城市核心竞争力，在此基础上向外拓展，发展黔北城市群。加强城镇规划建设和管理，加快实施中心城区道路交通规划，着力改善路网结构，提高保畅能力。加快撤县设区（市）步伐，力争遵义县、桐梓县、绥阳县、湄潭县撤县设区（市）。大力推进以县城、建制镇为重点的小城镇建设，着力培育一批综合型、加工型、商贸型、旅游型特色小城镇，建成一批10万人以上的中小城市。加大经济适用房和廉租房建设力度，调整住房供应结构，促进房地产业持续健康发展。加强城市综合执法，实施“数字化城管”、人性化城管，提高城市管理和服务水平。积极探索城市和城镇基础设施特许经营办法，推进市政公用事业市场化、产业化运作。巩固创卫成果，建立长效机制，提升城市品位；深化“四民社区”创建，努力提高市民素质，加强“整脏治乱”，推进城中村改造和“村改居”工作，不断改善人居环境，促进人才、管理、技术、资金、物流等各种要素向城镇汇聚，到2011年城镇化率达41%，力争创建国家环保模范城市和全国文明城市。

加强科技进步和技术创新，努力增强创新能力。认真实施市中长期科学和技术发展规划纲要，坚持自主创新、合作创新、引进消化吸收再创新“三位一体”的创新思路，加强知识产权保护，积极推进知识产权工作体系建设。加强以企业为主体、市场为导向、产学研相结合的区域创新体系建设，增强自主创新能力。依托061等大中型企业较强的科技研发能力，搭建技术创新“孵化器”平台，加快科技成果转化。大力发展技术咨询、评估、转让等中介服务机构，培育发展技术市场，建立较为完善的科技创新服务体系。加大科技投入和政策支持力度，形成多元化科技投入格局。抓好重点产业、资源循环利用、清洁生产以及关系人民群众健康安全等关键技术的研发，着力解决制约发展的科技问题，力争科技对经济社会发展的贡献率提高到46%。全面开展创建学习型机关、企业、社区、家庭等活动，构建全方位、多层次、开放式的全民学习、终身教育体系。在全社会培育创新意识，倡导创新精神，提高全社会参与创新的能力。完善创新机制，鼓励创新、宽容失败，形成崇尚创新、支持创新、追求创新的社会氛围，让创新的源泉充分涌流。

继续深化改革和扩大开放，努力激发创新活力。进一步深化改革，消除创新体制机制障碍。推进农村综合配套改革，完善农村税费改革各项配套措施，健全村民自治制度和农民负担监督管理制度。深化粮食流通体制改革，加强粮油流通市场调控，确保粮食安全。深化国有企业改革，加快建立现代企业制度，促进所有制结构调整。完善国有资产监督管理体制，推进国有资产的合理流动，盘活存量，扩大增量，做大总量。继续深化集体企业改革，发展多种形式的集体经济。加快城市公用事业改革和社会化服务体系建设，积极推进城镇管理、投融资体制、社会事业等改革，使各方面的创造才能得以发挥，发展愿望得以实现。

深化财税金融体制改革。完善市对县、区（市）的财政管理体制，加大对财政困难县的转移支付和强县强镇的扶持力度，充分调动各地生财聚财的积极性，大力培植税源，不断壮大财政实力；加快公共财政体系建设，优化支出结构，增加对“三农”和教育、公共卫生、公共安全、公益文化、社会保障等公共事业投入，确保重点支出；进一步加强预算管理，深化部门预算、国库集中收付、政府采购、收支两条线管理和政府收支分类改革，加快收入分配制度改革，积极推进公务和职务消费货币化改革；依法加强税收征管，规范税收征纳行为；推行财政支出绩效评价制度，提高财政资金使用效益；完善政府债务偿债机制，防范和化解财政风险；加强金融服务，改善金融生态环境，加强金融监管，积极防范和化解金融风险；深化农村金融改革，完善农村金融服务体系，规范发展保险业和证券业。

进一步扩大开放。全面放开基础设施、基础产业和制造业，加快推进金融、保险、信息咨询、现代物流、旅游等服务贸易领域对外开放，加快形成全方位、宽领域、多层次开放格局。坚持“紧贴黔中经济圈，融入长江经济带，参与泛珠经济区”， 加强区域经济合作与交流，发展外向型经济，不断增强遵义作为区域辐射受体和辐射中心的作用。依托重庆和贵阳资金、技术、信息、市场等优势，深化与两市在交通、工业、旅游、文化等方面的合作与交流，建设黔渝经济走廊。加大招商引资力度，提高对外贸易水平，力争全市招商引资到位资金年均增长15%，进出口贸易总额年均增长12%。

加快发展非公有制经济，增强经济发展活力。实施“中小企业成长工程”，消除体制障碍，放宽市场准入，扩大投资领域，完善服务体系，切实解决融资担保、技术转让、管理、人才培训、技术交流等方面的问题。鼓励中小企业与大企业、大项目配套协作，形成骨干企业带动、中小企业配套发展的产业集群。切实改进政府监督管理，强化公共服务，大力营造平等参与、公平竞争、宽松有序、依法经营、诚实守信的创业环境，促进各类所有制企业共同发展，掀起全民创业热潮，使非公有制经济成为经济增长的新动力源。到2011年，非公有制经济占生产总值的比重提高到50%以上。

（四）以建设文化旅游强市为重点，以构建新型服务业体系为核心，加快第三产业发展

坚持把旅游业作为第三产业发展重点，不断深化“中国优秀旅游城市”内涵，努力营造全社会发展旅游、服务旅游的良好氛围，打造文化旅游强市，进一步提高第三产业对经济增长的贡献率。

着力构建旅游综合服务体系，打造文化旅游强市。认真实施《遵义市旅游发展总体规划》和“八个一”工程，加强旅游交流与协作，积极推进川黔渝旅游“金三角”开发建设，尽快融入红色旅游经济圈。依托中国优秀旅游城市品牌，大力实施旅游精品带动战略，精心打造一批精品旅游景区和景点，重点培育以遵义会议会址、娄山关、四渡赤水为代表的红色旅游和以赤水、习水、绥阳、湄潭、余庆为代表的生态旅游，积极发展国酒文化旅游和以海龙囤为代表的地域特色文化旅游，加快开发以大乌江风景区为代表的东部、东北部生态旅游、民族文化旅游、湄潭浙大西迁文化旅游和茶文化旅游，把遵义建设成为全国红色旅游之城、休闲度假胜地。完善服务功能，改善服务环境，建设一批星级宾馆和酒店，加快旅游公路沿线配套服务设施建设；建设旅游特色街区，发展旅游特色城镇，开发旅游特色商品，打造地方特色美食；改革和完善旅游管理体制，组建旅游集团公司，推进点、线、面结合，优势互补，利益共享，大力扶持旅游企业发展，着力培养一批旅游服务人才，规范旅游市场秩序，形成功能齐全、文明卫生、服务周到、安全便捷的旅游环境，力争2011年全市接待游客1300万人（次）以上，年均增长18%，旅游综合收入达到88亿元，年均增长20%，把旅游业培育成为新兴的支柱产业。推进旅游产业与文化产业共同发展，整合教育、文化、广播电视、体育等各类文化资源，研究、发掘、提升物质文化和非物质文化遗产，加大包装、宣传、推介力度，大力弘扬遵义会议精神、长征精神，赋予新的时代内涵，振兴黔北文化，打造遵义人文精神。

着力构建新型服务业体系，提升服务业整体水平。推进服务业企业建立健全现代企业制度，促进服务领域社会化、产业化、市场化；充分发挥市场配置资源的基础性作用，打破服务业垄断经营，建立有利于市场公平竞争的资源配置机制，鼓励社会资本参与服务业发展；积极有序推进服务业对外开放，吸引国内外知名服务企业入驻遵义发展；引进现代化服务项目、经营管理模式和国际先进服务技术标准，提高服务业整体水平；积极运用现代经营方式和信息技术改造提升商贸、食宿、公交、公用事业等传统服务业，推动连锁经营、特许经营、物流配送、代理制、多式联运、电子商务等新型服务方式加速发展；加快发展现代物流、科技文化、金融保险、信息、中介、会计、设计、房地产、法律服务、咨询服务、社区服务、医疗保健、体育健身等新兴服务业。力争到2011年,服务业增加值占生产总值的比重达到40%以上。

（五）以基础设施建设为重点，以信息化为支撑，为跨越式发展提供基础保障

加强综合交通运输体系建设和公路管养。构建综合交通运输体系，大力实施“四纵三横三联二环”骨架公路网规划，加快南白至扎佐（遵义段）、白腊坎至茅台、杭州至瑞丽（遵义段）、马场坪至遵义（遵义段）、仁怀至赤水、中心城区环城高速公路，习水至桐梓新站、湄潭至余庆、绥阳至道真、务川接杭州至瑞丽（遵义段）、桐梓新站至绥阳太白等二级公路和崇遵高速公路娄山关匝道建设，实现县县通高等级公路；继续推进农村公路建设，全面实现乡乡通油路、村村通公路；继续抓好赤水河、乌江航道升级改造和构皮滩电站过船设施建设，切实改善水上交通条件；争取启动川黔铁路复线建设，尽快启用新舟机场，力争开工建设茅台机场。推进农村公路管养体制改革，加强农村公路管养，实现农村公路可持续发展。

加强水利基础设施建设。加大骨干水利工程、大中型灌区和烟（粮）水配套、雨水集蓄利用等“三小”水利工程建设力度,完成遵义灌区一期、湄凤余灌区二期和绥阳后水河灌区工程，完成遵义县下坝水库、湄潭关坎脚水库前期工作，力争启动正安石峰水库灌区一期、道真大沙河水库、遵义中桥水库等重点水利工程；积极推进节水灌溉工程建设，全面启动农村饮水安全工程。抓好农田灌溉工程建设，确保人均有效灌溉面积达到0.5亩。大力推进农村中小水电开发。创新水利管理体制机制，多渠道筹措资金，加大病险水库治理力度，每年完成60座以上治理任务。着力抓好中心城区、县城、乡镇供水工程改（扩）建和防洪工程建设，加大防灾减灾工作力度，基本形成防洪抗旱减灾体系和城乡水资源供给安全保障体系。

加强信息化基础设施建设。以电子政务先行，加快“数字遵义”建设，加强长途传输通信骨干网和数字通信网络建设，推进电信、有线电视、计算机三网融合，构建覆盖全市的公共信息传输网络。加快实施宽带智能网络工程，构建新一代多媒体网络平台。大力推进经济和社会信息化，不断提高工业生产数字化、智能化水平，带动电子制造业发展。全面推进信息技术在农业、服务业等经济领域和教育、卫生、文化、科技等社会领域的广泛应用，积极推进电子商务和企业信息化建设。

（六）以统筹协调发展为重点，以民生保障为核心，努力构建和谐遵义

加强环境治理保护，促进人与自然和谐。大力实施环境立市战略,努力建设资源节约型、环境友好型社会。建立环境保护和经济发展综合决策及考核机制，严格执行环境影响评价制度，推行环保一票否决制。建立健全资源开发有偿使用制度和生态恢复治理补偿机制，推进重点矿区生态恢复治理，切实加强饮用水源保护和农村面源污染防治，启动农村环保小康行动计划；加快中心区南部区域污染企业“退二进三”步伐。加大环境保护和污染治理力度，着力抓好重点区域、流域和行业的污染防治，强化对大气污染、水污染、固体废物污染和噪声污染的综合治理。实行减排目标责任制，确保主要污染物排放总量控制在省下达计划指标以内。进一步完善人口与计划生育利益导向机制，稳定低生育水平，人口自增率控制在6‰以内，总和生育率控制在1.69以内。加强流动人口管理，强化优质服务，加强出生人口性别比失衡的干预，提高人口素质，全面提升计生工作水平，促进人口、资源、环境协调。

统筹区域协调发展，促进区域发展和谐。支持西片区（仁、习、赤）壮大酿酒、能源、造纸、天然气化学工业，建成全市重要工业基地；大力发展红色旅游、生态旅游、国酒文化旅游、乡村旅游，打造知名旅游品牌；加快发展竹业、药业，建成特色农产品基地，推动西片区加速发展。支持中片区（两城区、遵、桐、绥）做大做强能源、原材料、冶金、有色、机电、建材、食品工业，大力发展高新技术产业，发展商品贸易和现代服务业，打造以“遵义会议会址”为核心的红色旅游品牌，加速南白—绥阳—桐梓循环经济重化工、铝钛工业基地建设以及桐梓河水电开发，实现中片区跨越式发展。支持东片区（湄、凤、余、正、道、务）加快“东部开发”步伐，大力实施《务正道凤片区开发规划》，加强交通、水利基础设施建设，消除东片区发展“瓶颈”制约；支持发展优质粮食、油料、烤烟、畜牧、中药材、茶叶等优势特色农产品及加工业，建成主要农特产品基地；加快乌江、芙蓉江、洪渡河水能资源开发和务正道铝土矿同步开发，建成重要的能源和铝工业基地；积极发展茶文化、民族民间文化和生态旅游，逐步把旅游业培育成支柱产业，实现东片区快速崛起。加大扶贫开发力度，积极化解贫困地区财政债务，争取和用好贫困地区、民族地区的扶持政策，逐步减少一类贫困乡镇。继续推进强县强镇建设,使之成为县域经济发展的“火车头”。

高度重视保障民生，促进社会发展和谐。继续实施积极的就业政策，鼓励全社会创业，大力发展劳动密集型产业、服务业和中小企业，多形式多渠道开发就业岗位，加大就业培训，千方百计扩大就业，优先促进“零就业家庭”就业。推动就业服务向社区和镇村延伸，积极探索城乡统筹就业新途径。到2011年，向非农产业和城镇转移农村富余劳动力35万人。加快建立新型劳动关系，严格执行最低工资制度。健全城镇养老、医疗、失业、生育、工伤保险和最低生活保障制度，推进农村社会保障制度，到2011年，城镇和农村参加社会保险人口分别占城镇和农村劳动者的70%和20%，城镇职工基本养老保险、失业保险、基本医疗保险覆盖率分别达75%、60%和80%以上。完善城乡一体化社会救助体系，加大城乡低保经费投入，提高保障标准。大力发展社会福利和慈善事业，深入开展“一帮一”、“送温暖”、“真情助学”等活动，着力解决特困群众住房、医疗、子女入学等问题。

以创建“平安遵义”为载体，全面落实社会治安综合治理措施。完善社会利益、纠纷调处机制，畅通群众利益诉求渠道，高效处理和妥善化解人民内部矛盾，预防和减少群体性事件；全面加强应急管理，提高预防和处置突发公共事件能力；坚持“安全第一、预防为主、综合治理”的方针，强化安全监管，切实保障人民群众生命和财产安全；认真实施“五五”普法规划，深入推进依法治市，加强法制宣传教育、人民调解和法律援助；进一步完善打防控一体化工作机制，依法严厉打击违法犯罪，促进社会治安进一步好转；认真贯彻落实党的民族、宗教政策，妥善处理好民族、宗教关系，切实解决少数民族聚居区发展滞后问题，促进各民族共同繁荣发展。

大力发展社会事业，促进经济社会和谐。优先发展教育事业。巩固提高“两基”成果，基本实现“双高普九”目标。加快发展高中阶段教育，力争中心城区和部分县市实现“普十二”目标。大力发展职业教育，构建与劳动就业紧密结合的新型职业教育体系，着力建设县、区（市）职教中心。整合教育资源，优化教育结构，积极筹建综合性遵义大学。加大教育投入，积极化解教育负债。完善农村义务教育经费保障新机制，加大对贫困家庭学生资助力度。注重发展幼儿教育和特殊教育，鼓励和支持民办教育，加快发展现代远程教育，促进各级各类教育协调发展。大力实施人才强市战略，认真组织实施“工程，力争人才总量和质量有较大突破；加快人才市场建设，建立健全运行规范、服务周到、指导监督有力的人才市场体系；坚持育人与用人相结合，用好现有人才，引进急需人才，培养后备人才；推进人才管理体制和机制创新，营造良好用人环境，激励各类人才充分发挥才智。

加快发展文化事业和文化产业。加强基础设施建设，努力构建公益文化服务体系，全面完成县（市）“两馆”建设，基本完成乡镇综合文化站新建改建任务，修建市群众艺术馆和文工团大楼，启用市图书馆和博物馆。加强文化遗产保护和利用工作，抓紧进行“海龙囤”、“四渡赤水战役旧址”、“湄潭浙江大学旧址”等全国重点文物保护规划的制定和申请立项维修工作，切实做好非物质文化遗产和历史文化名村、名镇的申报。不断挖掘文化资源，打造文化精品，加强和谐文化建设，经常开展群众性文化活动，充分发挥群众文化活动凝聚人、感染人、激励人的作用。扎实推进“万村书库”、“全国文化信息资源共享”、“农民文化家园”和“农家书屋”试点工程。积极开展对外文化交流和宣传活动，鼓励和支持多元化资本投入文化建设，大力扶持娱乐、演出、发行、影视传媒、新闻出版、印刷、网络文化、文化创意等文化产业，推动文化产业市场化、文化产品商业化。加强文化市场监管，促进文化市场健康发展。创新文化管理体制和工作机制，整合文化资源。建立经常性文化工作财政经费预算制度，确保文化工作扎实有效开展。

大力发展卫生等社会事业。坚持公共医疗卫生的公益性质，加快建立覆盖城乡居民的基本卫生保健制度，加强卫生基础设施建设，完善公共卫生和城乡医疗服务体系和卫生监督体系建设。普及新型农村合作医疗，建立和完善贫困群众医疗救助体系，有效解决群众看病难、看病贵问题，实现城乡居民人人享有基本医疗卫生保健服务。加强食品药品监管，确保群众用药饮食安全。扎实推进广播电视 “村村通”工程，加速广播电视数字化网络化进程。加快城乡公共体育设施建设，广泛开展全民健身运动。积极发展竞技体育，推进体育社会化、产业化，力争把遵义建设成为全国体育训练、竞赛的重要基地。加强国防后备力量建设和国防教育，巩固“全国双拥模范城”创建成果。积极发展气象、档案、编制、方志、人防、残疾人等社会事业，加强精神文明和民主法制建设，促进经济和社会协调发展。

（七）以构建公共服务型政府为目标，切实加强政府自身建设，推进政府管理创新

转变职能，建设服务政府。继续深化行政管理体制改革，推进政企、政事、政资分开，切实解决政府越位、缺位和错位问题，在履行好经济调节和市场监管的同时，切实加强社会管理和公共服务。创新公共服务体制，改进公共服务方式，优化公共资源配置，加强公共设施建设，提高社会管理水平。进一步完善政务服务中心运行机制，深化行政审批制度改革，减少审批事项，规范审批行为；加强效能建设和公务员队伍建设，健全行政监督、效能监察、考核奖惩机制，提高机关服务质量和效率；扩大政务公开，加快电子政务建设，完善政府综合门户网站，努力为社会提供透明、便捷的政务服务；完善政府新闻发布制度，提高政府工作透明度，保障群众的知情权、参与权和监督权。

依法行政，建设法治政府。加强社会主义民主政治建设，健全民主制度，丰富民主形式，扩大公民有序的政治参与。加强社会主义法制建设，深入贯彻国务院《全面推进依法行政实施纲要》，完善行政复议、行政赔偿等制度，加大行政综合执法、相对集中行政执法处罚权的改革力度，全面实施行政执法责任制和评议考核制度，加强行政执法监督，强化行政责任追究，提高行政执法水平。坚持依法决策、依法管理和依法办事，规范政府行为，自觉接受人大及其常委会的法律监督和工作监督，认真办理人大代表议案、建议；自觉接受人民政协的民主监督，大力支持人民政协履行职能，认真办理政协提案，主动加强与各民主党派、工商联、人民团体的联系；深入推进行政决策机制建设，建立健全重大决策专家咨询、顾问制度，重大事项社会公示和听证制度，重要会议邀请人大、政协参加制度，不断提高政府依法、民主、科学决策水平。

强化落实，建设实干政府。认真贯彻落实科学发展观，正确处理全局利益与局部利益、当前发展与长远发展的关系，增强大局意识，狠抓落实，确保政令畅通；严格目标责任制，抓重点、抓关键、抓调度，创新督查督办工作机制；完善绩效考评制度，严格行政问责，有责必问，有错必纠，建设责任政府；弘扬求真务实精神，大兴调查研究之风，深入矛盾最为集中、困难最为突出、群众最为需要的地方，了解实情、体察民意、研究对策、解决问题，充分调动好、保护好、发挥好基层干部干事创业的积极性；牢记“两个务必”，大力精简会议、文件，改进会风、文风，严格控制和规范各种检查、评比、达标活动，厉行节约，反对奢侈浪费，建设节约型机关。

强化监督，建设廉洁政府。树立“八荣八耻”的社会主义荣辱观，按照标本兼治、综合治理，惩防并举、注重预防的要求，加快建立健全教育、制度、监督并重的惩治和预防腐败体系，加强对权力运行的监督，建立健全决策、执行、监督既相互协调又相互制约的体制机制，做到靠制度管人，用制度管权，按制度办事。坚持不懈地开展反腐倡廉，推进源头治腐，加强重点行业、重点单位、重点部位的监管，加强政府采购、执法监察、效能监察，切实纠正部门和行业不正之风，切实解决人民群众反映突出的“热点”、“难点”问题；全面落实党风廉政建设责任制，严格执行廉洁从政规定，筑牢拒腐防变的思想防线；坚决从严治政，强化行政监察和审计监督，严厉查处违法违纪违规案件，全力塑造清正廉洁的政府形象。

三、2007年政府的主要工作

2007年是实施“十一五”规划纲要的重要之年，主要预期目标是：地区生产总值确保增长12.6%，力争达到14%；规模工业增加值增长16%以上；全社会固定资产投资确保增长15%，力争达到20%；社会消费品零售总额增长14%以上；财政总收入确保增长13%，力争达到15%；地方财政收入确保增长13%，力争达到16%；城镇居民可支配收入和农民人均纯收入分别增长8%以上；人口自增率控制在6‰以内；城镇登记失业率控制在4.6%以内；单位生产总值综合能耗下降4%以上，主要污染物排放减少2%。

围绕上述目标，2007年着重抓好八项工作：一是加快新农村建设，着力壮大农村经济。加强农业基础设施建设，进一步提高农业综合生产能力，完成粮食产量320万吨，烤烟收购170万担，农业增加值增长6%以上。推进农业结构调整，大力实施“六个一百”工程，加快发展劳务经济，促进农民增收，深化“四在农家”创建，促进农村面貌进一步改观。二是坚持走新型工业化道路，着力加速工业化进程。做强优势支柱产业，加快工业结构调整，降低能耗，提高工业发展质量和效益。赤水20万吨竹浆林纸一体化建成投产，遵义80万吨氧化铝建设完成计划进度，开工建设习水二郎火电厂、桐梓火电厂、桐梓煤化工、绥阳氯碱循环经济重化工基地，加快海绵钛、合金钢丝项目和铝、镁、锰等新材料基地建设;加快药业示范区和中药材基地建设及GAP认证工作；抓好茅台酒厂、遵义卷烟厂等企业技改。加强电煤保障和电、油、运等生产要素的协调调度，确保实现规模工业增加值增长目标。三是大力培育创新型城市，着力增强发展活力。继续深化各项改革，完善体制机制，增强发展活力。创新城市建设和管理，提升城市核心竞争力。完成中心城区建设投资10亿元、小城镇建设投资22亿元，房地产投资32亿元，城镇化率提高2个百分点。深入开展整脏治乱专项行动，巩固“创卫”成果，启动全国文明城市、国家环保模范城市创建活动，提高城市管理水平。四是加快培育文化旅游强市，着力扩大消费需求。继续实施“八个一”工程，加快旅游配套设施建设，确保旅游综合收入51亿元以上；加快构建新型服务业体系，确保服务业增加值增长13%以上，非公有制经济增加值增长20%以上。五是发挥投资拉动作用，着力促进投资合理增长。完成固定资产投资268亿元；续建和开工建设公路2195公里，完成交通建设投资27亿元以上。招商引资到位资金90亿元以上。六是狠抓开源节流，着力做好财税金融工作。巩固壮大骨干财源，培植新兴财源；加强税收征管，实现应收尽收；推进财政改革，调动各级各部门开源节流、增收节支的积极性；加强财政支出管理，提高财政资金使用效果；高度重视金融生态环境建设，实现地方经济与金融共同发展，银企互利双赢。七是全面发展社会事业，着力构建“和谐遵义”。高度重视民生，切实解决群众关心的热点、难点问题；统筹区域协调发展，促进区域经济各展所长；实施科教兴遵和人才强市战略，促进教育、科技、文化、卫生、计生等社会事业全面协调发展。八是推进政府职能转变和管理创新，进一步提高政府工作水平。

认真办好“十件实事”。1、完成娄山关景区陈列馆、停车场、步道及环境整治；完成习水、仁怀红军四渡赤水纪念地配套设施建设；2、建成红花岗公园；3、实施中心城区洛江河、忠庄河污水截流工程，实施中心城区供水管网配套工程，改扩建管网17.32公里；4、建成20万平方米经济适用住房（其中廉租房4万平方米），面向社会无房户、住房困难户、低收入群体销售;5、建设通乡油路200公里、通村公路1500公里，建成32个乡镇汽车客运站；6、实施农村贫困人口以工代赈易地扶贫搬迁工程，确保年内搬迁安置375户／1500人，同时实施农村危房改造工程2000户;7、新增“四在农家”创建点惠及14万户65万人，新建农村基层组织活动场所130个，改扩建121个;新建小水窖（池）1万口、生态沼气池10万口，解决8.95万人／4万头农村人畜饮水困难;8、改造和建设农村义务教育薄弱学校100所;9、开工建设乡镇卫生院55个，确保完成并投入使用44个，新型农村合作医疗覆盖500万农民;建设3个县计生服务指导站，确保年内主体工程完工;10、继续实施有线电视数字整体平移工程，新增数字电视用户14万户，建设广播电视“村村通”工程3000座。

各位代表，未来五年，是遵义全面实现“十一五”规划目标，谋求发展新跨越的五年，是全面建设小康社会，构建和谐社会的关键五年。让我们紧密团结在以胡锦涛同志为总书记的党中央周围，高举邓小平理论和“三个代表”重要思想伟大旗帜，全面落实科学发展观，在中共遵义市委的领导下，紧密团结和依靠全市各族人民，奋力拼搏，励精图治，加快发展，为建设经济繁荣、政治文明、文化发展、社会和谐的新遵义而努力奋斗！
